# How Stressors and Facilitators of Work–Family Dynamics Interrelate and Affect Psychological Outcomes in Farming Women: A Mixed-Methods Approach in Chile

**DOI:** 10.3390/healthcare13141760

**Published:** 2025-07-21

**Authors:** Gloria Mora-Guerrero, Fernanda Herrera-González, Carolina Alveal-Álamos, Jorge Constanzo-Belmar, Luis Marileo, Andrés Macadoo, Sharon Viscardi

**Affiliations:** 1 Escuela de Psicología, Facultad de Humanidades, Universidad de Santiago de Chile, Santiago 9170197, Chile; gloria.mora@usach.cl; 2 Programa de Doctorado en Planificación Territorial y Sustentabilidad, Universidad Católica de Temuco, Campus Luis Rivas del Canto, Rudecindo Ortega 03694, Temuco 4813302, Chile; 3 Departamento de Psicología, Facultad de Ciencias de la Salud, Universidad Católica de Temuco, Temuco 4813302, Chile; 4 Escuela de Medicina Veterinaria, Facultad de Recursos Naturales y Medicina Veterinaria, Universidad Santo Tomás, Temuco 4813302, Chile; 5 Biotechnology of Functional Foods Laboratory, Camino Sanquilco, Parcela 18, La Araucanía, Padre Las Casas 4850827, Chile; 6 Centro de Investigación, Innovación y Creación (CIIC), Universidad Católica de Temuco, Campus Luis Rivas del Canto Rudecindo Ortega 03694, Temuco 4813302, Chile; 7 Laboratorio de Investigación Interdisciplinaria en Microbiología Aplicada, Departamento de Procesos Diagnóstico y Evaluación, Facultad de Ciencias de la Salud, Universidad Católica de Temuco, Campus San Francisco, Manuel Montt 56, Temuco 4813302, Chile

**Keywords:** work-family interface (WFI), rural mental health, sociocultural mediators, gender-based dynamics, community facilitators

## Abstract

**Background/Objectives:** This study aimed to explore how stressors and facilitators within the work–family interface (WFI) influence mental health outcomes among farming women in rural Chile. The research sought to identify key relational patterns and contextual determinants shaping psychological well-being in this population. **Methods:** An exploratory mixed-methods design was employed, involving 41 semi-structured interviews analyzed using grounded theory. Qualitative themes were quantified by calculating the percentage of occurrence per interview, allowing for comparative analysis. Pearson correlation and principal component analysis (PCA) were used to examine associations among WFI dimensions and mental health-related variables. **Results:** Strong and statistically significant correlations emerged between institutional and community facilitators (r = 0.664, *p* < 0.01) and between gender facilitators and family workload stressors (r = 0.609, *p* < 0.01). PCA revealed two distinct patterns: women who rely on institutional support often resist traditional family roles, while others find balance through familial support systems. The gendered distribution of caregiving and productive tasks was a key factor in psychological well-being, with some women reporting physical discomfort linked to triple workloads. **Conclusions:** The dynamics of the WFI in rural contexts are shaped by both sociocultural and institutional factors. The findings highlight the need for culturally sensitive mental health policies that acknowledge and respond to the lived experiences of farming women.

## 1. Introduction

Rural populations encounter distinct mental health challenges, which have become increasingly significant due to the global deterioration in health conditions within these demographic segments [1,2,3]. These communities are undergoing profound sociocultural and socioeconomic transformations that substantially affect their psychological well-being, including elevated rates of depression and suicide [4,5,6]. Given the circumstances, there is a critical need to analyze the specific vulnerabilities of these populations to develop effective interventions aimed at improving their mental health [2,7].

However, despite the growing body of literature on rural mental health, there is a significant research gap concerning the specific psychological experiences of farming women in Chile. Although national reports acknowledge the challenges rural populations face, they rarely disaggregate data by gender or occupation, overlooking the intersectional burdens borne by women in agricultural households. This study addresses this critical knowledge gap by focusing on the mental health outcomes of farming women in rural Chile—an underexplored population whose well-being is shaped by overlapping stressors associated with caregiving, productive labor, and limited institutional support. Understanding their experience is essential for designing effective, culturally relevant mental health interventions in Latin American rural settings.

Mental health outcomes refer to the psychological consequences that emerge from sustained exposure to stressors and the availability or lack of facilitators in daily life. In rural and farming contexts, these outcomes often include emotional exhaustion, depressive symptoms, chronic stress, and anxiety [4,8,9]. For farming women, psychological well-being may also manifest as a sense of pride, resilience, and satisfaction derived from contributing to household economies and caregiving roles [7,10]. These outcomes are shaped by intersecting factors such as gender-based labor expectations, institutional access, and the cultural significance of agricultural roles. Recognizing these trajectories is essential to identifying both risk and protective elements that influence women’s capacity to sustain psychological health in rural settings.

There is some ambiguity in the studies on mental health status in rural contexts. The high rural suicide rate contrasts with generally more positive scores on mental state [11]. Some studies suggest that being born and residing in rural areas may be a protective factor when compared to growing up in urban environments, where psychiatric disorders such as schizophrenia, autism, substance abuse, and neurotic disorders are more prevalent [12,13,14,15]. However, this could be attributed to the agrarian cultural patterns that tend towards non-reporting and a greater focus on physical rather than mental health, leading to underdiagnosis [16].

While results differ based on the type of agricultural production, research trends generally indicate that members of farming families encounter significant mental health challenges. These challenges stem from pervasive risk factors associated with rural life, such as economic insecurity, social isolation, unpredictable weather conditions [17,18], and difficulties accessing mental health services [6,9]. Moreover, the extensive working hours required for farming, harvesting, and distribution exacerbate these challenges [19] and result in an elevated time pressure, all contributing to a distinctive stress associated with agricultural work [20].

Specifically for farming women, daily activities encompass both occupational and domestic tasks within a single physical space that also serves as a transgenerational heritage property, significantly influencing spatial identity and sense of place [10], where the work rhythm on the farm and various duties are aligned with family life [21,22]. This blurring of boundaries between work and family life exposes women to family relationships characterized by high job pressures added to economic insecurities inherent to agricultural activities [23]. Their inability to separate work from life creates persistent occupational stress [18], mainly because of their dual role in both an occupational and domestic sphere. Scholars agree that the persistence of these agricultural working conditions and poor work–life balance could aggravate women’s mental health issues.

We consider that one practical approach to addressing the work–family relationship and its implications on farming women’s mental health is to apply the insights obtained from the work–family interface (WFI) model. This model explores the interaction between the work and family domains, showing how these two aspects of life bidirectionally influence each other, impacting well-being [24,25,26]. The work–family interface (wFi) framework [25] serves as a central lens in this study. WFI explores how demands and resources from work and family domains interact to produce either conflict or enrichment. Conflict arises when pressures from one domain hinder participation in the other, while enrichment reflects positive transfers of energy, skills, or affect. In farming contexts, the boundaries between work and family are often blurred, making the WFI framework especially relevant. Additionally, spillover–crossover models [27,28] provide an important complementary perspective. These models distinguish between intra-individual processes (spillover), where stress or emotions transfer from one life domain to another within the same person, and inter-individual processes (crossover), where such effects impact closely related others. In rural households, these dynamics are intensified due to shared living and working environments, extended kinship roles, and multigenerational expectations.

Given the limited resources in terms of time, energy, and attention, naturally, the various roles assumed in different domains by women can come into conflict.

The literature identifies several negative consequences for women associated with the dynamics of the work–family interface, including life dissatisfaction and work-related stress [27,29]. The stress, strain, and burnout generated in the workplace impact family relationships, while conversely, family responsibilities can also conflict with work demands for women. Scholars predominantly explain these dynamics using the spillover–crossover model [28]. The spillover effect describes the intrapersonal process, where an individual’s experiences, emotions, skills, and behaviors in one life domain, such as work, are transferred to and influence another domain, such as home. In contrast, the crossover effect describes the interpersonal process where an individual’s experiences, emotions, or psychological states impact a significant other across different domains [30,31].

The WFI model additionally distinguishes between stressors and facilitators associated with each domain and the consequences they can have on people’s mental health. Stressors may include work and family commitments, job dissatisfaction, job and parental overload, a perception of increased skill discretion or decision-making authority at work, and the responsibilities associated with being married [32]. Regarding consequences, work and family stressors contribute to decreased organizational commitment, reduced job performance and stability, a significant risk for excessive alcohol consumption [33], and harmful psychological mechanisms such as sabotage and a poor ability to manage stress, which can lead to deteriorating mental and physical health [34,35,36]. On the other hand, influential factors such as supervisors, coworkers, and family support can act as facilitators in managing work–family dynamics, which could have positive consequences for people’s health [7,37]. Thus, although there is a strong theory about WFI dynamics, much less empirical evidence has been raised from the rural context.

A unique scenario emerges where the work and family domains are intricately linked, as in the farming household context. This scenario is an excellent opportunity to explore the potential for work–family enrichment through ‘role enhancement,’ wherein resources from one role may or may not bolster another [38]. This is especially true for adult farming women who often find themselves embedded in work–family norms with a significant gender-based basis. Rural sociocultural spaces preserve traditional forms of sexual division of labor, whereby women face unique challenges, as well as time constraints due to domestic work, caregiving tasks, and the triple shift [7,39,40]. The effects of emotional and mental overload associated with both caregiving and productive work and its implications for women’s psychological well-being have been documented [41,42,43], as well as their high rates of depression linked to family conflicts and limited social support [8,44]. Because this scenario is further intensified by the deterioration of their mental health conditions, in parallel with the notable socioeconomic and cultural transformations in the rural landscape, there is a demand for more knowledge about new challenges for attending to the rural mental health of women.

We attend to this knowledge demand by focusing on Chile, a country in the Latin American context where there is a recognized sense of belonging and social identity, as well as social support within the community-based territorial relationships [45,46,47,48], and where rural changes over the past decades have nonetheless led to increased stress levels among rural populations, in particular for women, who have also advocated for building new familial arrangements. This puts them in a complex social and familial position because, as they try to become more involved in paid work, they have less time to fulfill their traditional domestic and caregiving roles. Therefore, when people fail to meet their social expectations, a ‘work–family image discrepancy’ occurs, highlighting the gap between idealized roles and the challenging realities they face [5]. In terms of the psychological consequences of this, depression, heightened anxiety, and an increased rate of suicide have been attributed to male farmers [49]. Still, there is no clear evidence about the situation for women.

Introducing sociocultural mediators, such as territorial, gender, and family organization patterns, adds significant complexity regarding how the work–family relationship is experienced inside farming families [17,50]. In this context, the purpose of this study was to assess how the stressors and facilitators of work–family dynamics interrelate and affect mental health based on the experiences of farming women in Chile. Using a mixed-methods approach applied to farming women, we examine the stressors and facilitators that shape their work–family interface experience, as well as the consequences of these on their psychological well-being. This study aims to provide a nuanced understanding of how rural women navigate the challenges of work–family interrelationships and identify key areas for potential mental health interventions targeted at women who work in small-scale farming.

## 2. Methods

### 2.1. Study Location

This study was conducted in the La Araucanía region, located in southern Chile. The region accounts for approximately 5% of the national population, with nearly 30% of its inhabitants living in rural areas [51]. It is one of the poorest regions in the country and presents structural deficits in infrastructure and service access. In rural areas, one in three households lacks access to potable water, and only one in ten has a fixed internet connection [51]. Despite these limitations, rural communities in La Araucanía show strong organizational participation, with involvement in water committees (18.2%), Indigenous associations (13.9%), and religious groups (10.8%) [51]. The region also concentrates 916,933 hectares used for agricultural and forestry activities, representing 20.5% of Chile’s total agricultural area. The main crops include industrial crops (38.4%), cereals (35.3%), and produce from home gardens (31.4%) [51]. Within this context, family farming is the dominant productive and reproductive system. It is typically managed by the head of the household and relies primarily on family labor, often on small plots ranging from 2 to 5 hectares [52]. These demographic, socioeconomic, and cultural characteristics make La Araucanía a particularly relevant setting for studying how rural women navigate work–family dynamics and mental health challenges.

### 2.2. Study Design

The research presented here is part of a larger project examining how work–family dynamics are experienced by farming women in Chile. This study followed an exploratory QUAL–quant convergent mixed-methods design, in which qualitative data collection and analysis served as the foundation for a subsequent quantitative component. Reporting adhered to the Mixed Methods Reporting in Rehabilitation and Health Sciences (MMR-RHS) checklist, ensuring methodological transparency and integration between qualitative and quantitative strands. This design enabled the triangulation of findings and strengthened the explanatory power of the results by linking lived experiences with quantifiable relational structures.

Qualitative methods were prioritized to center the lived experiences of farming women, whose realities are often underrepresented in standardized surveys and formal registries. The use of semi-structured interviews allowed for an in-depth exploration of subjective meanings, sociocultural norms, and contextual stressors that shape work–family dynamics in rural Chile. Once thematic saturation was achieved, a quantitative strategy was introduced to systematically detect patterns of convergence and interrelation across cases. This sequential rationale ensured that the quantitative analysis was firmly grounded in context-specific narratives, enhancing both the explanatory power and relevance of the findings.

The results presented in this article are based on the quantitative analysis of primary data collected throughout the research project, which was conducted between 2019 and 2022. The study was organized into three main phases: an initial exploratory phase (March–November 2019); an intensive data collection phase (March–November 2020); and a final phase (March 2021–October 2022) focused on gathering specific data, validation, and follow-up. This last phase included interviews aimed at confirming emerging categories and triangulating findings with rural technical and community experts.

Semi-structured interviews were the main data collection technique used with farming women, complemented by field notes. Rural technical and community experts were also engaged during the process. The aim was to explore various dimensions of work–family balance and conflict and to obtain comprehensive data on the stressors, facilitators, and consequences of the triple shift carried out by women (productive, domestic, and social roles).

### 2.3. Sample and Data Collection

We conducted a total of 76 semi-structured interviews with diverse participants, including women involved in family farming as well as rural technical and community experts. For this article, we selected only the interviews with women actively engaged in family farming, yielding a final sample of 41 interviews that met the following inclusion criteria: (a) active participation in family farming activities, (b) performance of the role of housewife, and (c) residence within the study area. Five interviews conducted later in the research period, aimed primarily at validating preliminary findings, were excluded from this analysis (see Table 1).

For the purposes of this study, the role of housewife was defined as the primary responsibility for unpaid domestic and caregiving tasks within the household, including food preparation, cleaning, child-rearing, and/or elder care—regardless of whether the woman also engaged in productive farming or salaried work.

Semi-structured interviews were conducted in person by trained field researchers, either in participants’ homes or in community spaces. During the COVID-19 pandemic, some interviews were carried out by phone or conducted on-site while adhering to social distancing measures to prevent infection. Each interview lasted approximately 60 min. Audio recordings were transcribed verbatim and analyzed using open and axial coding, following grounded theory procedures. Thematic saturation was monitored throughout the fieldwork, and inter-coder reliability was ensured by double-coding a subset of transcripts.

To capture the diversity of rural women’s experiences, we employed maximum variation sampling [53], selecting participants based on variations in community type, economic workload, and caregiving responsibilities. Participants were then classified into four **worker identity** categories, adapted from Dunne et al. [54] and expanded in this study, according to how each woman recognized and described the value of her labor:

- **Traditional Housewives** (*n* = 4; 9.8%): Women exclusively engaged in domestic and caregiving work, without involvement in farming tasks, or who did not consider such activities part of their role.

*Example*: A woman who spends her day on household chores and child or elder care, without participating in crop or livestock activities.

- **Working Farm Family Members** (*n* = 4; 9.8%): Women who performed both domestic and productive tasks but considered their agricultural contributions to be informal support for male relatives, not part of the household’s formal economic activity.

*Example*: A woman who helps with harvesting or feeding animals but refers to it as “helping my husband” and does not view herself as a farmer.

- **Worker Farmers** (*n* = 19; 46.3%): Women who actively recognized their role in both productive and reproductive labor and considered their work essential to the household’s livelihood.

*Example*: A woman who manages planting, harvesting, and farm finances while also being responsible for cooking and caregiving.

- **Salaried Workers** (*n* = 14; 34.1%): Women who combined family farming with external paid work, such as employment in food services, cleaning, seasonal labor, or public programs.

*Example*: A woman who works three days per week in a school cafeteria and also maintains a home garden and household responsibilities.

Two members of the research team independently classified participants into the worker identity categories based on the qualitative indicators of self-recognition present in the interview transcripts. Specifically, self-recognition was assessed by analyzing participants’ own descriptions of their work, the terminology they used to define their economic role (e.g., “I only help”, “I bring money to the household”, “I work just like my husband”), and their perceived value of domestic versus productive contributions. These self-assessments were triangulated with the types of tasks they reported performing and how they framed those tasks in relation to household decision-making and income generation.

To ensure reliability in the classification process, both researchers coded each transcript independently and then compared their assigned identity labels. Inter-rater agreement was calculated using Cohen’s kappa, yielding a value of κ = 0.89, indicating strong consistency in the categorization process [55]. Any discrepancies were resolved through discussion and consensus, with reference to the original transcripts. All participants performed some form of productive work in addition to domestic tasks. However, their level of recognition of that work as economically or socially meaningful varied considerably. This subjective dimension of identity was critical in understanding how stressors and facilitators within the work–family interface were experienced and narrated.

### 2.4. Quantitative Design

The quantitative strand of this study aimed to transform rich qualitative data into a format suitable for statistical analysis. All interviews were transcribed and thematically coded using a grounded theory approach, based on a previously published framework of stressors, facilitators, and mental health outcomes [7]. For each interview, we calculated the percentage occurrence of each code by dividing the number of coded segments per theme by the total number of coded segments. This process yielded a proportional dataset that allowed us to perform a statistical analysis at the case level. Pearson correlations were conducted to explore thematic associations, and principal component analysis (PCA) was used to visualize convergence patterns among variables related to the work–family interface and mental health. We used Pearson’s correlation to examine thematic convergence, and we conducted principal component analysis (PCA) using PRIMER E-6 to visualize the patterns in the work–family dynamics and mental health outcomes. Table 2 shows the codes applied. These codes were identified and reported previously in a scientific article to describe the WFI model in farming families from women’s perspectives [7]. Prior to conducting Pearson correlation analyses, assumptions were assessed to ensure validity. All included variables were continuous and derived from proportional frequency data based on the standardized coding of interview themes. The normality of distributions was visually inspected through histograms and Q-Q plots, and no major deviations from linearity or homoscedasticity were observed. Given the standardized percentage format and the absence of extreme outliers, the assumptions for Pearson correlation, continuous data, linear relationship, and approximate normality were considered reasonably met.

To convert qualitative themes into quantitative data, each transcript was coded according to a predefined thematic framework derived from grounded theory (see Table 2). A coding unit was defined as a thematically distinct segment of text, such as a sentence or short paragraph, clearly associated with one of the identified stressors, facilitators, or consequences. For each interview, the total number of coded segments was counted, and the frequency of each theme was expressed as a proportion of that total. This yielded a matrix in which each case (interview) contained a set of percentage values representing the relative prominence of each theme within the participant’s narrative. For example, if a participant’s transcript had 50 coded segments and 10 of those were coded as “gender stressors”, the percentage occurrence for that code in that case would be 20%. This normalization allowed comparison across interviews of varying length and depth.

Thematic coding was conducted manually using a structured coding matrix shared between the research team in a document-based environment. No commercial qualitative software tools were used. Two researchers performed the coding process independently and applied the predefined framework line by line. The final matrices were compared and reviewed to ensure consistency in thematic application and segment boundaries. Any discrepancies were resolved through consensus discussions. This manual, collaborative process ensured the transparency and replicability of the conversion method and reinforced the validity of the quantitative analyses.

All interviews were conducted by a team of trained field researchers with prior experience in qualitative methods and rural contexts. Interviewers received a protocol outlining specific topics to explore, ethical considerations, and field procedures. Training sessions included mock interviews, cultural sensitivity guidelines, and procedures for informed consent. To ensure consistency across interviewers, regular debriefing meetings were held during the data collection period to discuss interview dynamics, clarify protocol application, and identify potential sources of interviewer bias. Inter-coder reliability checks were also applied during the coding phase, as described above, to align thematic interpretation across transcripts.

### 2.5. Ethical Considerations

Every participant was provided with an informed consent form previously approved by the host university’s Ethics Committee. In the informed consent, the research team committed to ensuring the confidentiality of the participants’ information and the anonymity of their names and other personal details to protect their privacy. This study adhered to ethical standards throughout the data collection and analysis process, ensuring respect for the dignity and rights of all participants. Materials and analysis codes for this study are not available, according to informed consent that authorizes the handling of participant information only by members of the research team.

## 3. Results

### 3.1. Significant Thematic Convergences and Correlations

Pearson correlations reveal moderately significant relationships that provide a deeper understanding of the dynamics of the work–family interface and its impact on the mental health of rural women. Table 3 shows the main results. The analysis showed the highest correlation between Institutional Context Facilitators and Community Facilitators (r = 0.664, *p* < 0.01), Institutional Context Stressors and Community Facilitators (r = 0.650, *p* < 0.01), Gender Facilitators and Family Farming Workload Organization Stressors (r = 0.609, *p* < 0.01), Psychological Consequences on Balance and Gender Facilitators (r = 0.633, *p* < 0.01), and Working Farm Family Member and Worker Farmer (r = −0.618, *p* < 0.01).

**Figure 1:**
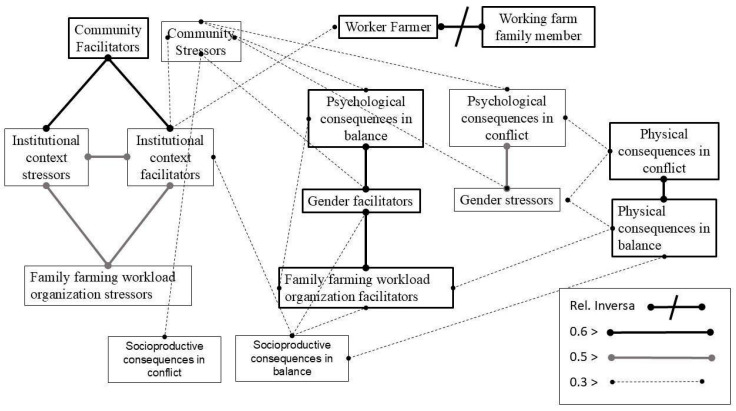
Thematic convergences and correlations in a global vision (Pearson).

The diagram in Figure 1 shows stratified correlations that provide a global vision. Correlations were classified based on their magnitude into three categories: strong (≥0.6), moderate (≥0.5 and <0.6), and weak (≥0.3 and <0.5) [56]. The visual representation differentiates these correlations using lines to indicate the varying strengths. This diagram facilitates the analysis of the relationship structure between categories, highlighting the following sets of variables.

The first set of categories indicates a strong positive correlation between the Gender Facilitators category and Psychological Consequences on Balance (r = 0.633) and with Family Farming Workload Organization Facilitators (r = 0.609). In another set of categories, the Community Facilitators variable showed a strong positive correlation with Institutional Context Stressors (r = 0.650) and Institutional Context Facilitators (r = 0.664). In a third set of categories, the Gender Stressors variable moderately correlates with Psychological Consequences in Conflict (r = 0.540). Finally, Worker Farmer identity correlates negatively with Working Farm Family Member (r = −0.618), while Physical Consequences in Conflict correlates positively with Physical Consequences on Balance (r = 0.667).

### 3.2. Principal Component Analysis Results

**Figure 2:**
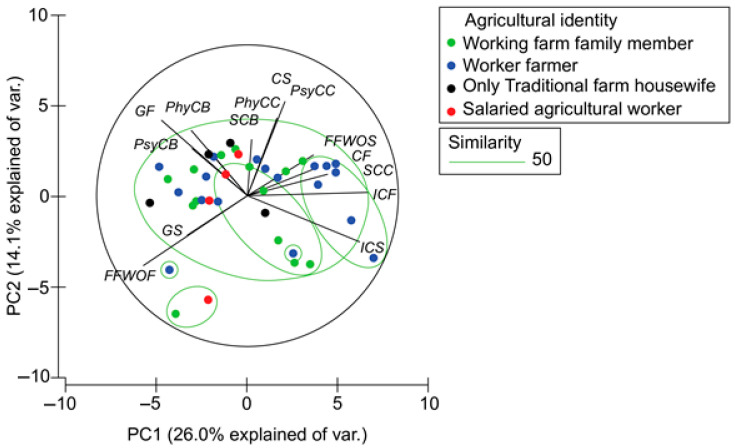
Principal component analysis results [57]. Generated with PRIMER v.6.

To explore the multivariate structure of relationships among work–family interface (WFI) themes and identity categories, we conducted a principal component analysis (PCA) (Figure 2). The analysis focused on standardized percentage values to visualize convergence patterns across interviews. Correlation strength thresholds were defined in advance following Schober et al. [56]: weak (r = 0.30–0.49), moderate (r = 0.50–0.69), and strong (r ≥ 0.70). These categories are used consistently in the interpretation of the correlation matrix (Table 3) and PCA findings.

The PCA extracted two main components: PC1, which explained 26.0% of the total variance, and PC2, which accounted for 14.1%. PC1 was shaped predominantly by institutional context facilitators (loading = 0.461) and institutional context stressors (loading = 0.441), while family farming workload organization facilitators loaded negatively (−0.400), reflecting a continuum from institutional support to reliance on internal family structures. PC2 was influenced by community stressors (loading = 0.444) and gender facilitators (loading = 0.382), forming a vertical axis from community-based pressure to gendered coping strategies.

These two axes intersect to define four quadrants in the PCA biplot:

- Quadrant I (PC1+, PC2+): High presence of institutional factors and community stressors.
- Quadrant II (PC1−, PC2+): Low institutional support, with reliance on gender facilitators and exposure to community stress.
- Quadrant III (PC1−, PC2−): Predominance of internal family strategies and minimal external support or stress.
- Quadrant IV (PC1+, PC2−): Greater access to institutional support, with fewer community-related challenges.

Each quadrant was associated with specific farming identity profiles. Quadrant I included several salaried workers who had access to formal training and welfare services but described emotional tension stemming from weak community ties or fragmented familial support. Although institutionally connected, these women navigated conflicting expectations between formal obligations and traditional rural roles.

Quadrant II primarily contained worker farmers who compensated for the absence of institutional support by relying on gendered strategies such as self-sacrifice and multitasking. Many of these women reported persistent exhaustion, time poverty, and emotional overload, despite recognizing themselves as key economic contributors to their households.

Quadrant III was composed mostly of traditional housewives and working farm family members who functioned within established family-based systems. These women exhibited limited interaction with institutions or community networks and often framed physical and emotional strain as a normalized part of their role, rarely articulating it as a mental health concern.

Quadrant IV included both worker farmers and salaried workers who reported alignment between institutional access and lower community stress. These women described greater autonomy, satisfaction with their productivity, and emotional balance. Their narratives reflected a shift toward more equitable household roles and recognition of their economic contribution.

These quadrant-based patterns illustrate how different combinations of institutional, familial, and community dynamics shape farming women’s experiences with the WFI. The results show that institutional engagement does not automatically reduce stressors if it is not supported by a change in household or community dynamics. At the same time, internal strategies may mitigate immediate tensions but often sustain unequal distributions of labor and emotional burden over time.

The associations revealed between identity categories and specific thematic patterns provide deeper insight into the ways work–family dynamics and mental health are interrelated in rural settings. These findings underscore the importance of designing mental health interventions that address not only individual experiences but also the structural, cultural, and relational conditions that shape the everyday lives of farming women.

### 3.3. Interpretation of Statistical Findings

The Pearson correlation analysis revealed strong and statistically significant associations that hold practical relevance for farming women’s mental health. For instance, the correlation between institutional and community facilitators (r = 0.664, *p* < 0.01) suggests that women who benefit from institutional support are also more likely to access community-based networks, reinforcing their overall resilience. Similarly, the significant association between gender-based facilitators and family workload stressors (r = 0.609, *p* < 0.01) highlights the double-edged role of gender roles: while some gendered capacities (e.g., multitasking) help women cope, they may simultaneously contribute to overload in traditional domestic expectations. These patterns were confirmed by the PCA, which showed that reliance on institutional support is often accompanied by a questioning of traditional family structures, whereas women embedded in strong familial networks tend to conform to established gender roles. These findings emphasize that mental health outcomes in rural women are not only shaped by individual coping skills but are strongly determined by the broader institutional and social environment.

## 4. Discussion

This section is organized along two analytical dimensions. The first addresses institutional and community facilitators and stressors, examining their interrelations and associations with family dynamics and women’s mental health outcomes. The second focuses on the relationship between family farming workload organization, gender-based dynamics, and psychological consequences. These two axes provide insight into how rural women’s well-being is shaped by both structural forces and intimate domestic arrangements.

### 4.1. Institutional and Community Facilitators/Stressors, Family Dynamics, and Women’s Psychological Well-Being

Our results reveal a strong interrelation between institutional facilitators and community-based networks. As shown in Figure 1, the presence of institutional resources—such as training, subsidies, or healthcare access—is positively associated with perceived support from community actors. This suggests a mutually reinforcing dynamic: institutional programs may gain legitimacy and effectiveness when channeled through local actors, while active community involvement may enhance women’s access to institutional pathways. This synergy may act as a protective factor for both physical and psychological well-being.

However, this relationship is not unidirectional nor universally beneficial. Principal component analysis revealed two divergent scenarios. In Quadrant I (Figure 2), we identified women with strong institutional access but high exposure to community stressors. Many of them reported emotional exhaustion, loneliness, or pressure to fulfill traditional expectations, despite having increased autonomy and economic opportunity. A possible explanation is that institutional participation may create tension with entrenched community norms or provoke conflict within households, particularly if women begin to question traditional roles. This echoes findings by Proctor and Hopkins [6] on the cultural dissonance experienced by women navigating shifting social roles in rural environments.

In contrast, Quadrant IV showed women with both institutional support and low exposure to community stressors, suggesting that alignment between public services and local dynamics can foster emotional balance and a greater recognition of women’s productivity. These results align with Putnik et al. [50], who emphasize the mediating role of sociocultural structures in work–family balance. Our findings indicate that when institutional facilitators are present and supported by community acceptance, women may renegotiate caregiving responsibilities, encourage male participation in domestic roles, and improve their psychological outcomes.

Importantly, we found a significant correlation between the “worker farmer” identity and institutional facilitators (Figure 1). Women who explicitly acknowledged their role in the economic sustainability of their households tended to access institutional resources more frequently and to perceive them as enabling tools rather than intrusive mechanisms. This supports the idea that identity recognition is not only a personal act but is shaped by the availability of structural support.

Nonetheless, institutional policies often ignore their impact on the micro-dynamics of rural households. Our findings point to the need to expand WFI frameworks to consider how institutional and community environments shape the invisible psychological burdens carried by women. While public programs often focus on economic indicators, they may inadvertently intensify emotional strain if not accompanied by efforts to shift cultural norms. As Sprung and Jex [38] suggest, economic stress can intensify work–family conflict unless addressed holistically.

### 4.2. Family Farming Workload Organization, Gender-Based Dynamics, and Psychological Consequences

Our data show that family farming workload organization interacts strongly with internal gender dynamics and mental health outcomes. When institutional facilitators were low, many women relied on internal family organization to maintain productivity, often under unequal gender arrangements. This was particularly evident in Quadrant II (Figure 2), where women described daily exhaustion, time poverty, and diffuse emotional distress, yet continued to perceive their experience as normative or even virtuous.

Family workload organization facilitators correlated directly with gender facilitators (Figure 1), indicating that in some households, cooperative arrangements and shared responsibilities mitigated emotional burden. Yet, for many, such facilitators remained aspirational. In the narratives of those embedded in traditional gender roles, caregiving, food provision, and field labor merged into a seamless continuum of obligations with few opportunities for rest or self-reflection.

Women often normalized symptoms such as fatigue, widespread bodily discomfort, or emotional numbness. This normalization may be rooted in the agrarian cultural model that defines work not merely as an economic activity but as a moral obligation. The family farm is often seen as a legacy, an identity, and a duty [10]. Vayro et al. [16] describe how agrarian values obscure the boundaries between care and labor, masking psychological strain behind the valorization of sacrifice. For rural women, this dynamic is further intensified by the spatial overlap between home and workplace, which erodes the possibility of psychological detachment from work demands.

Such invisibility may explain why mental health symptoms are underreported or reframed as physical ailments. The tendency to prioritize somatic over emotional complaints is well documented among rural populations [58], and our study confirms that gendered divisions of labor are a key driver of this phenomenon.

### 4.3. Implications of Gender Dynamics, Institutional Support, and Family Workload Distribution

The relationships between gender dynamics, institutional support, and family workload distribution appear central to understanding psychological outcomes. Women who expressed pride in their productivity and a sense of autonomy often reported access to supportive networks and greater participation in decision-making. Their narratives included references to training, the diversification of income, and shared domestic responsibilities—factors associated with positive psychological indicators (PsyCB).

Conversely, those who adhered to traditional workload patterns (FFWOF) commonly described chronic fatigue and vague psychological distress, often without identifying it as such. As one participant put it: “I don’t have time to rest; between the house, the field, and helping the community, I don’t stop until I go to bed.” This quote reflects the internalization of normative expectations that demand self-sacrifice and uninterrupted productivity.

Such findings underscore the dual nature of family roles: while they can be sources of meaning and cohesion, they can also perpetuate unequal burdens that compromise well-being. Women who rely exclusively on family structures without external support described feeling isolated and overwhelmed. Meanwhile, those with access to institutional programs often had more opportunities for self-care and emotional validation, reinforcing the importance of context-sensitive, intersectional approaches to rural mental health.

## 5. Limitations

This study is grounded in the sociocultural mediators’ framework [50], with a particular emphasis on gender. However, it does not incorporate an intersectional analysis that considers other dimensions such as ethnicity, socioeconomic status, educational level, or political participation [59,60]. Including these factors could deepen the understanding of how structural inequalities interact and shape rural women’s mental health and work–family dynamics. Similarly, the exclusion of other perspectives—such as those of children, adolescents [61], and men [62]—limits the scope of the findings, which focus solely on women’s narratives. Future studies could benefit from incorporating these viewpoints to obtain a more comprehensive view of family farming systems.

Additionally, this study does not engage in a comparative analysis of rural policies or regulatory frameworks, which could help contextualize the findings at the institutional level [63]. Another limitation lies in the use of self-reported data and the classification of participant work identities based on self-recognition. While inter-rater reliability checks were performed to reduce bias, the subjective nature of self-recognition may still influence the categorization and interpretation of certain results.

## 6. Conclusions

Current theories on WFI tend to focus too much on the individual, neglecting the broader context of institutional, community, and family organizations and gender roles. Our findings suggest that a more holistic approach is needed—one that incorporates sociocultural and family dynamics and gender as essential factors in understanding how women navigate the balance between work and family responsibilities and its psychological consequences. From a methodological perspective, this study presents a novel approach by applying a quantitative procedure to the data from the grounded theory-based interviews to convert the occurrence values of key themes (qualitative codes) into percentages, providing a comparison across interviews and applying Pearson’s correlation and principal component analysis (PCA).

Specifically, our study showed how farming women have developed traditional mental health practices deeply rooted in the community and familial organizations. We also learned how the factors identified play a positive or negative role in psychological well-being. However, more research is needed to clarify specific relationships between institutional, community, and familial contexts and women’s ability to improve their health. We encourage scholars to generate more scientific evidence, especially in Latin America, where social cohesion in rural communities can serve as a protective factor for women’s mental health [45,46,47,48], but also where recent sociocultural and economic transformations can weaken these support networks, increasing isolation and vulnerability [55,64].

The literature suggests the possibility that women’s greater institutional and community participation can imply a work overload [65,66], but our research shows contradictory evidence of this. Work overload can be an effect of gender-based inequalities inside families, even if these do not openly challenge inequalities within the private sphere. However, we agree with [11] in the sense that there is a latent dissatisfaction with this type of familial arrangement, which can lead to the underdiagnosis and undervaluation of psychological issues. Transgressing gender patterns can have familial and social disciplining effects on women, while reinforcing gender norms can lead to an experience of well-being in women, even if this keeps them in a situation of inequality. Educational programs that promote mental health awareness can empower women to identify and address psychological challenges more effectively. These findings call for a renewed understanding of rural women’s mental health, not as an isolated individual issue but as a reflection of deeply rooted structural dynamics within agricultural life. Recognizing the interdependence between institutional support, community cohesion, and family organization is essential for designing effective, context-sensitive interventions. Ultimately, promoting psychological well-being among farming women requires not only access to mental health services but also a transformation in how caregiving, productive labor, and gender roles are socially distributed and valued within rural territories.

## 7. Practical Implications

The findings of this study have direct implications for rural mental health policy and intervention design. First, recognizing the coexistence of caregiving, productive, and community roles in farming women underscores the need for integrated support systems that consider both institutional access and family dynamics. Programs aimed at improving women’s mental health in rural areas should move beyond individualized clinical approaches and incorporate culturally sensitive strategies that strengthen community networks and redistribute caregiving responsibilities within families. Moreover, this study highlights the value of promoting women’s participation in institutional programs not only as beneficiaries but also as agents of organizational change, which can foster more equitable gender dynamics. Finally, the use of a mixed-methods approach provides a replicable model for identifying invisible labor burdens and psychosocial stressors in similar rural contexts, informing evidence-based interventions that are both locally grounded and gender-responsive.

## Figures and Tables

**Table 1:** Sample profile by Worker Identity Group.

Worker Identity Group	*n*	% (of 41)
Traditional housewives	4	9.8%
Working farm family members	4	9.8%
Worker farmers	19	46.3%
Salaried workers	14	34.1%

**Table 2:** Analysis codes.

Work–Family Farming Interface		
Type	Description	Code
Stressors		
Community stressors	Social mistrust and lack of social and institutional support	CS
Family farming workload organization stressors	A change in the traditional farming workload organization, which implies an increased workload on a housewife (e.g., she was widowed or divorced, a child migrates or moves away, or a family member needs to be cared for)	FFWOS
Gender stressors	Deficient abilities for daily life associated with gender-based socialization (e.g., low technological proficiency and lack of skills to express opinions or discomfort or to defend their rights)	GS
Institutional context stressors	Limited access to technology, basic utilities, or services (e.g., drinking water, electricity, public transportation), and/or limited availability of public services (e.g., incompatible schedules)	ICS
Facilitators		
Community facilitators	Existence of community care networks (e.g., grandmothers or others), institutional networks (e.g., access to public services and trainings), and a sense of belonging in the neighborhood	CF
Family farming workload organization facilitators	A traditional family workload organization, where male partners and children share the workload in accordance with their gender and age	FFWOF
Gender facilitators	Abilities for daily life associated with gender-based socialization (e.g., spatial and temporal integration strategy)	GF
Institutional context facilitators	Access to basic utilities and technology, satisfactory public services (e.g., productive skills training)	ICF
Positive Health Consequences		
Physical consequences on balance	Exhausting journeys and hard labor perceived as part of a “normal” productive life	PhyCB
Psychological consequences on balance	A sense of pride in one’s job or ability to earn money, in being busy all day, and in building new opportunities for future generations	PsyCB
Socioproductive consequences on balance	A feeling of making the most out of one’s time (using time efficiently to complete a lot of tasks)	SCB
Negative Health Consequences		
Physical consequences in conflict	Feeling widespread pain and physical discomfort; feeling exhausted	PhyCC
Psychological consequences in conflict	Feeling emotional discomfort (anger, guilt, helplessness, disempowerment, monotony, or bad mood), feeling a lack of time (time poverty), and/or becoming a victim of gender-based violence	PsyCC
Socioproductive consequences in conflict	Difficulties in time management to access training or general services (e.g., health)	SCC

**Table 3:** Significant thematic convergences and correlations (Pearson).

Code	Code	r
Working Farm Family Member	Worker Farmer	−0.618
Family Farming Workload Organization Stressors	Community Facilitators	0.524
Institutional Context Facilitators	Community Facilitators	0.664
Institutional Context Stressors	Community Facilitators	0.650
Gender Facilitators	Family Farming Workload Organization Facilitators	0.609
Psychological Consequences on Balance	Gender Facilitators	0.633
Psychological Consequences in Conflict	Gender Stressors	0.540
Institutional Context Stressors	Institutional Context Facilitators	0.583
Physical Consequences in Conflict	Physical Consequences on Balance	0.667

## Data Availability

Data is contained within the article.

## References

[1] Saravanakumar P., Muhammad T., Paul R., Srivastava S. (2024). Explaining the Urban-Rural Difference in Late-Life Depression in India: Evidence from a Multivariate Decomposition Analysis Based on Longitudinal Aging Study in India, Wave 2017–18. Clin. Gerontol..

[2] Wiese L.A.K., Gibson A., Guest M.A., Nelson A.R., Weaver R., Gupta A., Carmichael O., Lewis J.P., Lindauer A., Loi S. (2023). Global rural health disparities in Alzheimer’s disease and related dementias: State of the science. Alzheimer’s Dement..

[3] World Health Organization (2021). WHO Guideline on Health Workforce Development, Attraction, Recruitment and Retention in Rural and Remote Areas.

[4] Brumby S., Chandrasekara A., McCoombe S., Kremer P., Lewandowski P. (2011). Farming fit? Dispelling the Australian agrarian myth. BMC Res. Notes.

[5] Ladge J.J., Little L.M. (2019). When Expectations Become Reality: Work-Family Image Management and Identity Adaptation. Acad. Manag. Rev..

[6] Proctor C., Hopkins N. (2023). Stressors and Coping Strategies in Rural Farmers: A Qualitative Study. J. Agromed..

[7] Mora-Guerrero G., Herrera-González F., Constanzo-Belmar J., Alveal-Álamos C., Viscardi S. (2023). Uncovering the Work–Family Interface: The Impact of Facilitators and Stressors on the Health of Farm Women. Healthcare.

[8] Letvak S. (2002). The importance of social support for rural mental health. Issues Ment. Health Nurs..

[9] Morales D.A., Barksdale C.L., Beckel-Mitchener A.C. (2020). A call to action to address rural mental health disparities. J. Clin. Transl. Sci..

[10] Ellis N.R., Albrecht G.A. (2017). Climate change threats to family farmers’ sense of place and mental well-being: A case study from the Western Australian Wheatbelt. Soc. Sci. Med..

[11] Chiswell H. (2023). Psychological Morbidity in the Farming Community: A Literature Review. J. Agromed..

[12] Amshoff S.K., Reed D.B. (2005). Health, Work, and Safety of Farmers Ages 50 and Older. Geriatr. Nurs..

[13] Peen J., Schoevers R.A., Beekman A.T., Dekker J. (2010). The current status of urban-rural differences in psychiatric disorders. Acta Psychiatr. Scand..

[14] Solmi F., Dykxhoorn J., Kirkbride J.B. (2017). Urban-Rural Differences in Major Mental Health Conditions. Mental Health and Illness Worldwide.

[15] Vassos E., Agerbo E., Mors O., Pedersen C.B. (2016). Urban–rural differences in incidence rates of psychiatric disorders in Denmark. Br. J. Psychiatry.

[16] Vayro C., Brownlow C., Ireland M., March S. (2020). ‘Farming is not Just an Occupation [but] a Whole Lifestyle’: A Qualitative Examination of Lifestyle and Cultural Factors Affecting Mental Health Help-Seeking in Australian Farmers. Sociol. Rural..

[17] Bragger J.D., Rodriguez-Srednicki O., Kutcher E.J., Indovino L., Rosner E. (2005). Work-family Conflict, Work-family Culture, and Organizational Citizenship Behavior Among Teachers. J. Bus. Psychol..

[18] Thompson R., Hagen B.N.M., Lumley M.N., Winder C.B., Gohar B., Jones-Bitton A. (2023). “An Incredible Amount of Stress before You Even Put a Shovel in the Ground”: A Mixed Methods Analysis of Farming Stressors in Canada. Sustainability.

[19] Brigance C., Soto Mas F., Sanchez V., Handal A.J. (2018). The Mental Health of the Organic Farmer: Psychosocial and Contextual Actors. Workplace Health Saf..

[20] Somashekar B., Reddy P.S., Wuntakal B., Chaturvedi S. (2020). Stress and Rural Mental Health. Mental Health and Illness Worldwide. Mental Health and Illness in the Rural World.

[21] Dedieu B., Contzen S., Nettle R., de Alencar Schiavi S.M., Sraïri M.T. (2022). The Multiple Influences on the Future of Work in Agriculture: Global Perspectives. Front. Sustain. Food Syst..

[22] Elliot V., Cammer A., Pickett W., Marlenga B., Lawson J., Dosman J., Hagel L., Koehncke N., Trask C. (2018). Towards a deeper understanding of parenting on farms: A qualitative study. PLoS ONE.

[23] Burgard S.A., Lin K.Y. (2013). Bad Jobs, Bad Health? How Work and Working Conditions Contribute to Health Disparities. Am. Behav. Sci..

[24] Frone M.R., Russell M., Cooper M.L. (1992). Antecedents and outcomes of work-family conflict: Testing a model of the work-family interface. J. Appl. Psychol..

[25] Greenhaus J.H., Beutell N.J. (1985). Sources of Conflict between Work and Family Roles. Acad. Manag. Rev..

[26] Yucel D., Fan W. (2019). Work–Family Conflict and Well-Being among German Couples: A Longitudinal and Dyadic Approach. J. Health Soc. Behav..

[27] Westman M., Jones F., Burke R.J., Westman M. (2006). Crossover of stress and strain in the work-family context. Work-Life Balance.

[28] Bakker A.B., Demerouti E., Grzywacz J.G., Demerouti E. (2013). The spillover–crossover model. New Frontiers in Work and Family Research.

[29] Chen Z., Hou L. (2021). An Actor-Partner Interdependence Model of Work Challenge Stressors and Work-Family Outcomes: The Moderating Roles of Dual-Career Couples’ Stress Mindsets. J. Bus. Psychol..

[30] Li A., Cropanzano R., Butler A., Shao P., Westman M. (2021). Work–family crossover: A meta-analytic review. Int. J. Stress Manag..

[31] Ratnaningsih I.Z., Idris M.A. (2024). Spillover–Crossover Effect of Work–Family Interface: A Systematic Review. Fam. J..

[32] Stoiko R.R., Strough J., Turiano N.A. (2017). Understanding “His and Her” Work-Family Conflict and Facilitation. Curr. Psychol..

[33] Calvo-Salguero A., Martínez-de-Lecea J.S., del Carmen Aguilar-Luzón M. (2012). Gender and work–family conflict: Testing the rational model and the gender role expectations model in the Spanish cultural context. Int. J. Psychol..

[34] Bakker A.B., de Vries J.D. (2021). Job Demands–Resources theory and self-regulation: New explanations and remedies for job burnout. Anxiety Stress Coping.

[35] De Gieter S., De Cooman R., Bogaerts Y., Verelst L. (2022). Explaining the effect of work–nonwork boundary management fit on satisfaction and performance at home through reduced time- and strain-based work–family conflict. Appl. Psychol..

[36] de Sousa C., Viseu J., Pimenta A.C., Vinagre H., Ferreira J., Matavelli R., José H., Sousa L., Romana F.A., Valentim O. (2024). The Effect of Coping on the Relationship between Work-Family Conflict and Stress, Anxiety, and Depression. Behav. Sci..

[37] Landini F., González-Cowes V., Leite J., Ferreira Leite J., Dimenstein M., Dantas C., Macedo J.P. (2021). Rural Psychology: Literature Review, Reasons for Its Need, and Challenges. Psychology and Rural Contexts.

[38] Sprung J.M., Jex S.M. (2017). All in the family: Work–family enrichment and crossover among farm couples. J. Occup. Health Psychol..

[39] FAO (2022). Enfoque de Género en Los Programas de Extensión Rural en Chile.

[40] Mora Guerrero G.M., Constanzo Belmar J.D. (2018). ‘Emprender sin descuidar la casa’: Posiciones y dinámicas organizativas en una asociación productiva de mujeres rurales. Cuad. Desarro. Rural.

[41] Gutiérrez V. (2020). Impacto de las múltiples crisis en la vida de las mujeres rurales. La Suma de Todas las Crisis y las Resistencias Feministas.

[42] Mascheroni P., Angulo S. (2022). Cuidar en el campo: Trabajo remunerado de cuidado en el Uruguay rural. Rev. Latinoam. De Estud. Rural..

[43] Parreira B.D.M., Goulart B.F., Ruiz M.T., Cristina dos Santos Monteiro J., Gomes-Sponholz F.A. (2021). Sintomas de ansiedade entre mulheres rurais e fatores associados. Esc. Anna Nery.

[44] Guruge S., Thomson M.S., George U., Chaze F. (2015). Social support, social conflict, and immigrant women’s mental health in a Canadian context: A scoping review. J. Psychiatr. Ment. Health Nurs..

[45] Contreras R. (2000). Empoderamiento campesino y desarrollo local. Rev. Austral De Cienc. Soc..

[46] Durston J. (2002). El Capital Social Campesino en la Gestión del Desarrollo Rural. Díadas, Equipos, Puentes y Escaleras.

[47] Guitart M., Sánchez Vidal A. (2012). Sentido de comunidad en jóvenes indígenas y mestizos de San Cristóbal de las Casas (Chiapas, México). Un estudio empírico. An. Psicol..

[48] Terry Gregorio J. (2012). Aproximación al concepto de comunidad como una respuesta a los problemas del desarrollo rural en América Latina. Contribuciones a las Ciencias Sociales.

[49] Hammersley C., Meredith D., Richardson N., Carroll P., McNamara J. (2023). Mental health, societal expectations and changes to the governance of farming: Reshaping what it means to be a ‘man’ and ‘good farmer’ in rural Ireland. Sociol. Rural..

[50] Putnik K., Houkes I., Jansen N., Nijhuis F., Kant I.J. (2020). Work-home interface in a cross-cultural context: A framework for future research and practice. Int. J. Hum. Resour. Manag..

[51] Ministerio de agricultura (2024). Ficha Regional, Región de La Araucanía.

[52] Biblioteca del Congreso Nacional de Chile (BCN) El rol de la mujer en la Agricultura Familiar Campesina. https://www.bcn.cl/obtienearchivo?id=repositorio%2F10221%2F34413%2F1%2FMinuta_70_23_El_rol_de_la_mujer_en_la_agricultura_familiar_campesina_FPHM_2023.pdf.

[53] Dahlgren L., Emmelin M., Hällgren Graneheim U., Sahlén K., Winkvist A. (2007). Qualitative Methodology for International Public Health.

[54] Dunne C., Siettou C., Wilson P. (2021). Investigating the economic visibility and contribution of UK women in agriculture through a systematic review of international literature. J. Rural. Stud..

[55] Landis J.R., Koch G.G. (1977). The measurement of observer agreement for categorical data. Biometrics.

[56] Schober P., Boer C., Schwarte L.A. (2018). Correlation Coefficients: Appropriate Use and Interpretation. Anesth. Analg..

[57] Sharma E., Das S. (2021). Integrated model for women empowerment in rural India. J. Int. Dev..

[58] Balakrishnan A., Ahluwalia H., Desai G., Chaturvedi S. (2020). Common Mental Disorders and Folk Mental Illnesses. Mental Health and Illness in the Rural World.

[59] Lagarde M., González M.L. (1996). Lagarde, M. La multidimensionalidad de la categoría género y del feminismo. Metodología Para Los Estudios de Género.

[60] Viveros Vigoya M. (2016). La interseccionalidad: Una aproximación situada a la dominación. Debate Fem..

[61] Vázquez Luna D., Mortera Pucheta D., Rodríguez Orozco N., Martínez Martínez M., Velázquez Silvestre M.G. (2013). Organización comunitaria de mujeres: Del empoderamiento al éxito del desarrollo rural sustentable. Velázquez Silv. María Gisela.

[62] Connell R.W., Valdes T., Olavarría J. (1997). La organización social de la masculinidad. Masculinidad/es: Poder y Crisis.

[63] Osorio C. (2011). La emergencia de género en la nueva ruralidad. Rev. Punto Género.

[64] del Mármol C., Vaccaro I. (2020). New extractivism in European rural areas: How twentieth first century mining returned to disturb the rural transition. Geoforum.

[65] Malapit H., Meinzen-Dick R.S., Suseela R., Quisumbing A.R., Zseleczky L. (2011). Women: Transforming Food Systems for Empowerment and Equity. 2020 Global Food Policy Report.

[66] Margolies A., Colantuoni E., Morgan R., Gelli A., Caulfield L. (2023). The burdens of participation: A mixed-methods study of the effects of a nutrition-sensitive agriculture program on women’s time use in Malawi. World Dev..

